# The Effects of Work Characteristics Related to Work–Life Imbalance on Presenteeism among Female Workers in the Health and Social Work Sectors: Mediation Analysis of Psychological and Physical Health Problems

**DOI:** 10.3390/ijerph18126218

**Published:** 2021-06-08

**Authors:** Jee-Hyun Hwang, Hye-Sun Jung

**Affiliations:** Department of Preventive Medicine, College of Medicine, The Catholic University of Korea, 222 Banpo-daero, Seocho-gu, Seoul 06591, Korea; hjh2830425@naver.com

**Keywords:** health and social work sectors, work characteristics, psychological health issues, physical health issues, presenteeism, work–life imbalance

## Abstract

This study is a multigroup path analysis aiming to create a theoretical model of presenteeism among female workers in the health and social work sectors, assess the mediating effects of mental health problems (sleeping trouble and psychological wellbeing), and physical health problems (fatigue and muscle aches) on the relationship between work characteristics (demands at work and social community at work) and presenteeism, and identify the differences between the effects of variables on those who reported low work–life imbalance and high work–life imbalance. Raw data from the fifth Korean Working Conditions Survey (KWCS) were analyzed. From the total sample of 50,205 people, 2209 women in health and social work were included in the study sample. The results were as follows: the demands at work had a significant and positive indirect effect on presenteeism (B = 0.0023, *p* < 0.001), mediated by trouble sleeping, fatigue, and muscle aches, in that order. Demands at work were also found to have a significant and negative indirect effect on presenteeism (B = −0.0017, *p* < 0.001), mediated by psychological wellbeing, fatigue, and muscle aches, in descending order. Social community at work had a significant and negative indirect effect on presenteeism (B = −0.0022, *p* < 0.001), mediated by trouble sleeping, fatigue, and muscle aches, in that order. Social community at work also had a significant and negative effect on presenteeism, mediated by psychological wellbeing, fatigue, and muscle aches, in descending order (B = −0.0097, *p* < 0.001). Demands at work did not have a significant effect on psychological wellbeing in the low work–life imbalance group, whereas its effect was significant and positive (*β* = 0.198, *p* < 0.001) in the high work–life imbalance group. In conclusion, in the path model of the low work–life imbalance group, demands at work did not influence psychological wellbeing. Therefore, strategies to ameliorate work–life imbalance may be helpful components of interventions to reduce presenteeism.

## 1. Introduction

Women’s increasing participation in economic activities and the growing social awareness of quality of life have been accompanied by an increasing interest in work–life balance among women. The popular conceptualization of work–life balance has recently changed to emphasize the balance between an individual’s entire work and non-work life, rather than solely focusing on the balance between work and family life [1]. Issues around work–life balance and imbalance can apply to both men and women, but familial pressure related to childbirth and child rearing is traditionally higher for women, which increases the burden of work–life imbalance on women [2].

Work–life imbalance has a negative effect on an individual’s physical and psychological health [1]. A study by Camerino et al. [3] found that work–life conflict had a significant association with presenteeism. Work–life balance has received considerable social attention, and was ranked second after salary in terms of its importance in people’s view of work [4].

Many companies are interested in promoting the physical and psychological health of workers in order to reduce absences and enhance the company’s productivity. This trend has led to an increased interest in work–life balance and presenteeism [5].

Presenteeism refers to the physical and psychological problems resulting from an individual going to work while sick. In other words, it denotes the act of going to work when a worker’s health status suggests that they should not [6]. Close attention must be paid to presenteeism, since the practice of going to work while sick can exacerbate a worker’s health problems and threaten future job prospects and quality of life [7]. Moreover, the negative effects of presenteeism on productivity have caused many companies to become interested in ways of addressing presenteeism. Generally, predictions of costs related to workers’ illnesses focus on estimating medical costs from sick leave or absence, disability due to accidents, and direct loss of work hours. However, these are only some of the elements related to decreases in productivity. In fact, indirect costs resulting from decreased work capacity due to presenteeism outweigh the costs resulting from absences [8]. Therefore, the importance of presenteeism from the perspectives of both individual workers and society underscores the need to pay close attention to this issue [4].

According to a study on decreases in productivity and work capacity due to presenteeism [9], the levels of presenteeism were higher in the nursing and social work sectors than in other work sectors. In these particular work sectors, relationships with other people play an important role in work results. Since workers in these sectors mainly care for sick people, children, and the elderly [9], the health of these workers not only concerns them as individual workers, but also affects the health of more dependent and vulnerable groups. Therefore, lax health management of these workers can diminish the quality of health and social welfare services, reduce organizational productivity, and, in some cases, cause medical errors and safety issues [5]. In South Korea, the Korean Working Conditions Survey (KWCS), which is conducted among a stratified and randomized sample of households, provides valuable information on the workforce. According to the KWCS, 13.4% of employees in the health and social work sectors are men, while 86.6% are women; thus, these sectors are occupations with a substantial majority of female workers.

Research on presenteeism among female workers in the health and social work sectors has mainly involved health problems, musculoskeletal pain, poor mental health, trouble sleeping, depression, and burnout [10,11,12,13]. Presenteeism was traditionally studied with a focus on individuals’ physical and psychological statuses, but, more recently, research has attempted to clarify the relationship between workplace characteristics, such as work demands, work environment, and workers’ levels of control and presenteeism [4,14,15].

Cooper [16] conceptualized six factors (work demands, control, social community at work, fairness, and value) that influence the mismatch between the job and the individual in the job–person mismatch model. Pohling [17] suggested that these factors can explain presenteeism. A greater mismatch between these six factors exacerbates workers’ psychological problems and physical fatigue, causing presenteeism.

Of the six factors that cause job–person mismatch, demands at work and social community at work have an important effect on workers’ psychological and physical health. Lee et al. [18] found that greater demands at work reduced workers’ quality of sleep, which is a physical health issue. A study by Choi [19] suggesting that social community at work has a significant effect on psychological wellbeing supports this finding. These findings may be due to the effects of demands at work and the deterioration of the social community on sleep problems and psychological distress. Poor sleep quality can also cause fatigue, since physical and psychological recovery becomes difficult [20].

A review of the literature on the work environment, work–life imbalance, psychological and physical health issues, and presenteeism demonstrated that studies on the associations between work–life balance and job satisfaction and between work hours and work–life imbalance constituted the majority of research [1,21], while studies that assessed the relationships among variables according to differences in work–life balance were lacking. There is a lack of studies on the associations between variables stratified by differences in work–life imbalance. Thus, it is necessary to consider differences in work–life imbalance and to conduct research on presenteeism as a factor mediated by workers’ psychological and physical health, which are products of one’s work environment.

In South Korea, the Korean Working Conditions Survey (KWCS) collects data for comprehensively assessing work environment. The KWCS, which was developed based on the European Working Conditions Survey, encompasses various areas, such as work demands, work organization, work content, cooperation and leadership, and work–life balance [19]. The job–person mismatch model, as presented by Shin [4], is an appropriate model in terms of methodology for analyzing secondary data from workers, since job–person mismatch is largely measured based on how individuals perceive and interpret situations. Most items for measuring the factors that influence presenteeism ask individuals to subjectively evaluate the environment and conditions of their workplaces, which is an appropriate method for measuring job–person mismatch [4].

Therefore, this study aimed to understand the impact of work–life imbalance on presenteeism as mediated by psychological and physical health issues among female workers in the health and social work sectors in South Korea using the fifth KWCS conducted in 2017. Based on the job–person mismatch model from a study by Cooper [16] and the factors that explain presenteeism in a study by Pohling [17], psychological and physical health issues related to presenteeism in the low work–life imbalance group and high work–life imbalance group were assessed using multigroup path analysis. Work–life imbalance was conceptualized as a mediator in the theoretical path model of this study based on a study by Yang et al. [22], which found that work–life balance influenced presenteeism in terms of subjective wellbeing rather than directly, and other related studies that used work–life balance as mediators [23,24,25].

The objective of this study was to identify and examine differences in the effects of work characteristics (demands at work and social community at work), psychological health issues (trouble sleeping, psychological wellbeing), and physical health issues (fatigue and muscle aches) on presenteeism according to the level of work–life imbalance.

The results of this study are expected to improve our understanding of presenteeism in female workers in the health and social work sectors and contribute to the establishment of effective human resources management policies for medical or social welfare organizations. Since presenteeism is an important social issue that has a greater negative effect on work capacity than productivity loss from the use of health services and absence from work [8], it is valuable to study presenteeism to promote the health of female workers.

## 2. Materials and Methods

### 2.1. Study Design

**Figure 1:**
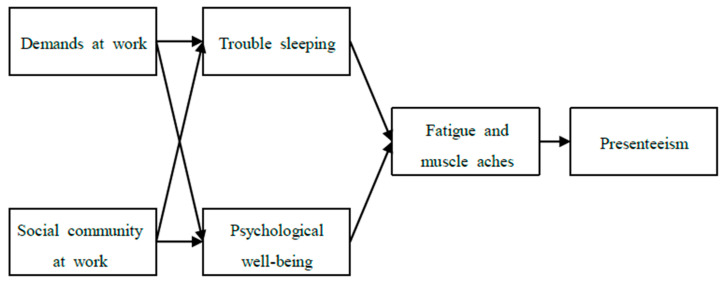
Conceptual framework for this study.

This multigroup path analysis study aimed to establish a theoretical model to examine presenteeism among female workers in the health and social work sectors, to understand the mediating effects of psychological health issues (trouble sleeping, psychological wellbeing) and physical health issues (fatigue and muscle aches) in the relationship between work environment and presenteeism, and to examine differences in those effects according to levels of work–life balance and imbalance (Figure 1).

### 2.2. Study Sample

This study used raw data from the fifth KWCS. From the total sample of 50,205 people, data on 2974 potential subjects who were in the health and social work sectors were extracted for this study. After excluding men, 2575 female participants remained. After excluding 366 people with missing variables, 2209 subjects were ultimately selected as the final study sample. According to occupational status, the final study sample consisted of workers at hospitals, clinics, public healthcare facilities, non-resident welfare facilities, and residential welfare facilities, as well as those who provided other healthcare services.

### 2.3. Measurements and Variable Definitions

#### 2.3.1. The Korean Working Conditions Survey (KWCS)

The survey was conducted from July to November 2017 to develop an overall understanding of different factors related to work environments, including work status, employment status, job types, job categories, exposure to risk factors, and safety of workers over the age of 15 in South Korea. The KWCS included 50,205 individuals who worked for more than 1 h in the previous week for income, including wage workers, business owners, and self-employed individuals. The survey was administered through a one-on-one interview conducted by a professional interviewer at a household visit.

#### 2.3.2. Work Characteristics

Work characteristics included demands at work and social community at work. Demands at work were measured with two items: “working very fast” and “having a tight work schedule due to deadlines.” Responses were recorded using a Likert scale, with a score of 1 indicating “always” and 7 indicating “not at all,” and the resulting scores were reverse-coded for analysis. A higher score indicated more demands at work. Regarding the tool’s reliability, the Cronbach’s ⍺ was 0.75 in June and Choi’s study [26] and 0.840 in this study. Social community at work was measured with three items: “I get proper recognition for my work,” “I generally get along with my coworkers,” and “the organization I work for motivates me to work at my best capability.” Responses were recorded using a Likert scale, with a score of 1 indicating “completely agree” and 5 indicating “completely disagree,” and the resulting scores were reverse-coded for analysis. A higher score indicated a greater sense of social community at work. Cronbach’s ⍺ was 0.66 in June and Choi’s study [26] and 0.724 in this study.

#### 2.3.3. Trouble Sleeping

Trouble sleeping was measured with three items: “difficulty falling asleep,” “waking up repeatedly while sleeping,” and “waking up exhausted or feeling extremely fatigued.” Responses were recorded using a Likert scale, with a score of 1 indicating “every day” and 5 indicating “not at all,” and the resulting scores were reverse-coded for analysis. A higher score indicated a higher degree of trouble sleeping. Cronbach’s ⍺ was 0.870 in Bae and Kim’s study [27] and 0.871 in this study.

#### 2.3.4. Psychological Wellbeing

The measurement tool for psychological wellbeing was the WHO-5 wellbeing index developed by the World Health Organization. This index is used to screen for psychological wellbeing and depression, has been studied in various fields [28], and includes five items: “I have felt cheerful and in good spirits,” “I have felt calm and relaxed,” “I felt active and vigorous,” “I woke up feeling fresh and rested,” and “my daily life has been filled with things that interest me.” Responses were recorded using a Likert scale, with 1 indicating “all the time” and 6 indicating “at no time,” and the resulting scores were reverse-coded for analysis. A higher score indicated better psychological wellbeing. Cronbach’s ⍺ was 0.93 in Kim’s study [29] and 0.911 in this study.

#### 2.3.5. Fatigue and Muscle Aches

Fatigue and muscle aches were assessed with one item: “do you have back pain, muscle pain in the upper body (shoulders, neck, arms, elbows, wrists, and hands), muscle pain in the lower body (glutes, legs, knees, and feet), headache or eye fatigue, or overall body fatigue?” Responses were either 1 for “yes” or 2 for “no.” “Yes” was coded as 1, and “no” was coded as 0. A higher score indicated more symptoms of fatigue and muscle pain. Cronbach’s ⍺ was not reported in Lee [30] and was 0.736 in this study.

#### 2.3.6. Work–Life Imbalance

When individuals cannot participate in their daily lives to their satisfaction as much as they participate in work, they experience a large work–life imbalance. Work–life imbalance was measured with five items: “I worry about work when I am not working,” “I cannot do household chores because I am too exhausted after work,” “the time I can spend with my family is not sufficient due to work,” “the time I can use to work is not sufficient due to family matters,” and “I feel I cannot put time into work due to family responsibilities.” Responses were recorded in a Likert scale, with 1 indicating “always” and 5 indicating “not at all,” and the resulting scores were reverse-coded for analysis. A higher score indicated greater work–life imbalance. Cronbach’s ⍺ was 0.84 in Hong’s study [21] and 0.882 in this study.

#### 2.3.7. Presenteeism

Although presenteeism can refer to the impact of workers going to work while ill on productivity, it is interpreted more broadly as the act of workers going to work despite being sick and being in a health condition that suggests they should miss work [6]. This study measured the number of days that individuals worked despite being sick with the item used by Kim [29], and considered presenteeism to be demonstrated when the number of days worked despite being sick was one or more. Presenteeism was determined using participants’ responses regarding the number of days they worked while sick, with a minimum possible number of days of 0 and maximum of 365.

### 2.4. Statistical Analysis

SPSS version 23 (IBM, New York, NY, USA) and AMOS version 20 (IBM, New York, NY, USA) were used to analyze the data for this study. In order to assess the reliability of the measurement tools, Cronbach’s α coefficient for each tool was determined. A descriptive statistical analysis was conducted to examine the sub-factors of participants’ psychological and social characteristics, such as demands at work and social community at work, psychological health issues (trouble sleeping and psychological wellbeing), and physical health issues (fatigue and muscle aches), in addition to presenteeism and the different levels of work–life imbalance. To examine the correlations between the main variables of this study, a Pearson correlation analysis was conducted. To assess the model fit constructed using AMOS 20 (IBM, New York, NY, USA), the Tucker–Lewis index (TLI), comparative fit index (CFI), and root mean square error of approximation (RMSEA) were calculated. The statistical significance of *β* values and *p*-values for each path coefficient was evaluated to examine the influence of each path on the others in the model. A bootstrap validation was performed to verify the indirect effects. Multigroup analysis was conducted to assess differences in the influences of variables on other variables according to the level of work–life imbalance. To confirm differences in the influences of variables on other variables according to the level of work–life imbalance, Δχ^2^ statistics and *p*-values were calculated. *β* values and *p*-values were assessed to determine the significance of each path after stratifying high and low work–life imbalance groups.

### 2.5. Ethical Considerations

This study was conducted after being approved by the institutional review board of the Catholic University of Korea (IRB; approval date: 26 February 2021) and performed in accordance with the Declaration of Helsinki.

## 3. Results

### 3.1. General Characteristics of the Subjects

For this study, 2209 female workers in the health and social work sectors were analyzed. A frequency analysis of the general characteristics of the study subjects showed that 27.4% were aged 40–49, 22.9% were aged 50–59, 21.8% were aged 30–39, 16.1% were aged 60 and over, and 11.8% were aged 20–29. Furthermore, 60.6% of the respondents indicated that they had a spouse, 39.0% indicated that they did not have a spouse, and 0.4% had no response. The most common level of education was high school or lower (37.6%), followed by university or higher (34.0%) and two-year college (28.4%). The household size was four people for 27.5% of the participants, two people for 27.1%, three people for 26.8%, one person for 12.8%, and five people or more for 6%.

### 3.2. Job-Related Factors of the Subjects

A frequency analysis was conducted to identify the job-related characteristics of the participants. The distribution of workplaces was as follows: non-resident welfare facilities, 51.2%; hospitals, 24.0%; clinics, 20.1%; residential welfare facilities, 3.0%; public healthcare facilities, 1.3%; and other healthcare services, 0.4%. Occupational status was paid employment for 96.5% of participants, self-employment for 3.2%, and other forms of employment for 0.3%. The majority of respondents (85.4%) reported that they did not engage in shift work, while 14.6% did. Slightly fewer than half of the respondents (48.5%) worked in small businesses (1–9), followed by 45.3% who worked in medium businesses (10–249), 5.8% in large businesses (250 or more), and 0.9% who did not respond.

### 3.3. Descriptive Statistics of Variables

A descriptive statistical analysis was conducted to measure the predominance of each of the main variables according to their respective scores from the measurement tools. Scores for demands at work ranged from 2 to 14, with a mean of 5.86, and they ranged from 3 to 15 for social community at work with a mean of 10.71. For trouble sleeping, scores ranged from 3 to 15 and had a mean of 4.60, while psychological wellbeing had a score range of 6–30 and a mean score of 20.02. Scores for fatigue and muscle aches ranged from 0–5 and had a mean of 0.82. To determine presenteeism, the participants supplied the number of days they worked while sick, with a minimum possible number of days of 0 and maximum of 300. The mean number of days was 1.03. The skewness and kurtosis of the presenteeism variable were very high, so the analysis was performed after log transformation of the variable. Work–life imbalance had scores ranging from 5 to 25, and a mean score of 11.20.

### 3.4. Correlations Between Variables

In order to examine the correlations between the main variables in this study, Pearson correlation analysis was conducted (Table 1).

### 3.5. Fitness of the Path Model

This study set psychological health issues (trouble sleeping and psychological wellbeing) as the first mediator and physical health issues (fatigue and muscle aches) as the second mediator on the effect of work characteristics (demands and social community at work) on presenteeism. To assess this model, the following path model was constructed.

To assess the fitness of the constructed model, the main goodness of fit indicators were calculated. A non-significant result for the chi-squared statistic indicates good model fit, but this statistic is sensitive to sample size. When the sample size is large, TLI, CFI, and RMSEA are used to determine the goodness of fit instead of the chi-squared statistic [31]. Therefore, goodness of fit was evaluated based on the TLI, CFI, and RMSEA in this study.

Generally, the model fit is considered good when the TLI and CFI are greater than 0.90 and the RMSEA is less than 0.08 [32]. For the path model in this study, TLI = 0.956, CFI = 0.982, and RMSEA = 0.034, indicating the goodness of fit of the study model.

### 3.6. Assessment of the Significance of Path Coefficients

To determine the impacts of paths on other paths in the model, the statistical significance of each path coefficient was assessed.

The path from demands at work to trouble sleeping was significant and positive (*β* = 0.219, *p* < 0.001), and the path from social community at work to trouble sleeping was significant and negative (*β* = −0.122, *p* < 0.001). The path from demands at work to psychological wellbeing was significant and positive (*β* = 0.096, *p* < 0.001), and so was the path from social community at work to psychological wellbeing (*β* = 0.314, *p* < 0.001). The path from trouble sleeping to fatigue and muscle aches was significant and positive (*β* = 0.136, *p* < 0.001), there was a significant and negative path from psychological wellbeing to fatigue and muscle aches (*β* = −0.231, *p* < 0.001), and the path was significant and positive from fatigue and muscle aches to presenteeism (*β* = 0.339, *p* < 0.001).

Trouble sleeping was higher when demands at work were high and social community at work was low. Psychological wellbeing was high when demands and social community at work were high, while fatigue and muscle aches were high when trouble sleeping was high and psychological wellbeing was low. Presenteeism was high when fatigue and muscle aches were high (Table 2).

### 3.7. Assessment of Indirect Effects

To assess indirect effects, bootstrap validation was conducted. The number of bootstrap samples was set at 20,000, and significance was measured at the 95% confidence level. In the results, the 95% confidence interval did not include 0 in the four indirect effect paths, indicating that the indirect effects were statistically significant.

Demands at work were mediated by trouble sleeping and fatigue and muscle aches sequentially, and had a significant and positive indirect effect on presenteeism (B = 0.0023, *p* < 0.001). Demands at work were also mediated by psychological wellbeing and fatigue and muscle aches sequentially, and had a significant, negative, and indirect effect on presenteeism (B = −0.0017, *p* < 0.001).

Social community at work was mediated by trouble sleeping and fatigue and muscle aches sequentially, and had a significant, negative, and indirect effect on presenteeism (B = −0.0022, *p* < 0.001). Social community at work was also mediated by psychological wellbeing and fatigue and muscle aches sequentially, showing a significant and negative indirect effect on presenteeism (B = −0.0097, *p* < 0.001) (Table 3).

### 3.8. Differences in the Influence of Variables According to the Level of Work–Life Imbalance (Multi-Group Analysis)

Using the median of work–life imbalance as a cutoff, subjects were classified into low and high work–life imbalance groups. To assess the differences in the influence of variables according to the level of work–life imbalance, a multi-group analysis was conducted. A model that restricted the paths to be the same between the low and high work–life imbalance groups and a model with no restrictions were compared.

The two models had a statistically significant difference (Δχ^2^ = 94.738, *p* < 0.001). This result suggests that there was a significant difference in the influence of variables according to the level of work–life imbalance. Therefore, in order to understand how the influence of variables differs, the significance of each path was evaluated separately for the low and high work–life imbalance groups.

The impact of demands at work on trouble sleeping was positive and significant in both groups. The effect was larger in the high work–life imbalance group (*β* = 0.222, *p* < 0.001) than in the low work–life imbalance group (*β* = 0.067, *p* < 0.05).

The impact of social community at work on trouble sleeping was negative and significant in both groups. The effect was larger in the high work–life imbalance group (*β* = −0.136, *p* < 0.001) than the low work–life imbalance group (*β* = −0.070, *p* < 0.05).

The impact of demands at work on psychological wellbeing was not significant in the low work–life imbalance group. However, the impact was significant and positive in the high work–life imbalance group (*β* = 0.198, *p* < 0.001).

The impact of social community at work on psychological wellbeing was significant and positive in both groups. The difference between the low (*β* = 0.342, *p* < 0.001) and high (*β* = 0.280, *p* < 0.001) work–life imbalance groups was not large. This finding suggests that psychological wellbeing was high when social community at work was high for both groups.

The impact of trouble sleeping on fatigue and muscle aches was positive and significant in both groups. The effect of trouble sleeping on fatigue and muscle aches was larger in the low work–life imbalance group (*β* = 0.213, *p* < 0.001) than in the high work–life imbalance group (*β* = 0.078, *p* < 0.01).

The impact of psychological wellbeing on fatigue and muscle aches was negative and significant in both groups. The effect of psychological wellbeing on fatigue and muscle aches was larger in the high work–life imbalance group (*β* = −0.271, *p* < 0.001) than in the low work–life imbalance group (*β* = −0.179, *p* < 0.001).

**Figure 2:**
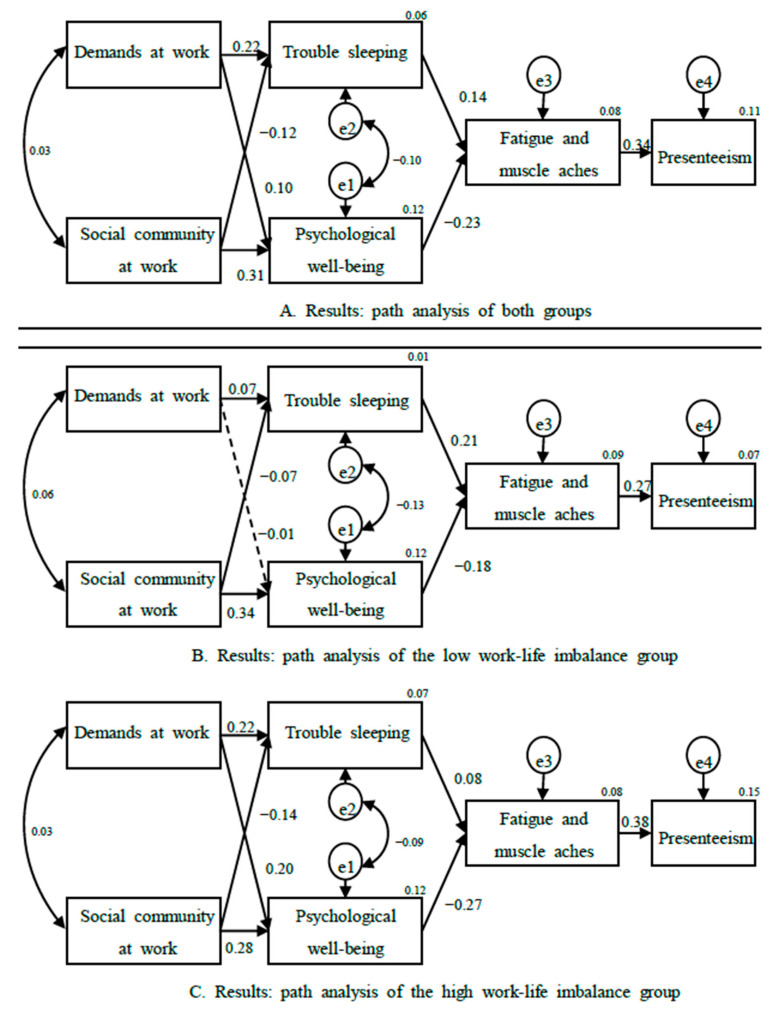
The results of the path analysis.

The impact of fatigue and muscle aches on presenteeism was positive and significant in both groups. The effect of fatigue and muscle aches on presenteeism was larger in the high work–life imbalance group (*β* = 0.381, *p* < 0.001) than in the low work–life imbalance group (*β* = 0.267, *p* < 0.001) (Table 4) (Figure 2).

## 4. Discussion

Women’s increasing participation in economic activities and the growing social awareness of quality of life have prompted increasing interest in work–life balance among women. Therefore, this study analyzed the influence of work characteristics (demands at work and social community at work) as they pertained to work–life imbalance on presenteeism among female workers in the health and social work sectors. In this process, multigroup path analysis was used to examine the mediating effects of psychological health issues (trouble sleeping and psychological wellbeing) and physical health issues (fatigue and muscle aches).

Trouble sleeping was high when demands at work were high and social community at work was low (due to factors such as an overall lack of motivation at the organization). It was reported that the quality of sleep of nurses in university hospitals was poorer when they had more work and their sense of social community at work was low, as indicated by a low reported level of mutual support from managers and coworkers (18). Therefore, efforts must be made to adjust individuals’ workloads appropriately in consideration of their work capabilities and to establish an organizational culture that can increase the sense of social community within the organization.

Psychological wellbeing was higher when demands at work and social community at work were high. Generally, psychological wellbeing decreases when work demands are high. In a study involving elementary school teachers, it was found that the risk of depression increased as work demands increased [33], but the results of this study differ from the results of other existing studies. The results of this study are similar to those of a study involving married nurses that found subjects to have a positive outlook, despite having a heavy workload [34]. It can be inferred that, when individuals feel pride as professionals, psychological stability increases, even with high work demands. However, in a study that examined the association between social community at work and depression among nurses using the same tool used in this study [19], depression was high when social community at work was low, which corresponds to the results of this study, showing low psychological wellbeing when social community at work was high.

Fatigue and muscle aches increased when trouble sleeping was high and psychological wellbeing was low. In a study involving nurses at a university hospital, sleep quality had an independent and major impact on fatigue [35]. Bliwise [20] also found that, when sleep quality was poor, fatigue was also high, since physical recovery became difficult. It has been shown that mental dissonance affects somatization symptoms, such as muscle aches and headaches in nurses [36], and Cooper [16] examined the effects of the deterioration of psychological wellbeing on fatigue and muscle aches by observing how psychological tension among workers increased the experience of fatigue. Therefore, the development of intervention programs and the establishment of effective management strategies that can reduce psychological health issues are necessary to prevent fatigue and muscle aches before they occur due to trouble sleeping and poor psychological wellbeing.

An analysis of the effect of fatigue and muscle aches on presenteeism revealed that presenteeism increased with fatigue and muscle aches. In a study involving clinical nurses, it was found that fatigue affected presenteeism and that, to prevent productivity loss, the management of fatigue is necessary for nurses [10]. Another report showed that musculoskeletal pain among physiotherapists affected presenteeism [11]. That report found that, in order to reduce losses in productivity by physiotherapists from presenteeism, it is necessary to manage musculoskeletal pain by improving the work environment to account for their work hours and work status [11]. Presenteeism is an important social issue that causes greater losses in productivity resulting from reduced work capacity than the productivity losses resulting from the use of health services and absence from work [8]. Therefore, it is necessary to design and implement human resource protocols for helping workers to manage physical health issues early, such as fatigue and muscle aches, and to establish an organizational culture in which time can be taken off from work at appropriate times.

When the multigroup path analysis results of the low work–life imbalance group were compared to those of the high work–life imbalance group, the effect of demands at work on trouble sleeping was greater in the high work–life imbalance group. Multiple studies found that quality of sleep decreased when demands at work increased [18,37]. For women, a high work–life imbalance may be partially due to the expectations placed on them from their families related to childbirth and child-rearing [2], which make it difficult to balance work and life. If demands at work increase at a time when work and life are already imbalanced, it can cause trouble sleeping. A previous study also reported that work–life conflict had a direct effect on sleep [38]. This finding supports the result of this study, which found that demands at work had a greater effect on trouble sleeping in the high work–life imbalance group.

The effect of social community at work on trouble sleeping was greater in the high work–life imbalance group. A study by Oh [38] reported that work–life conflict had a direct effect on sleep, supporting the results of this study, which found that social community at work had a greater effect on trouble sleeping in the high work–life imbalance group.

While demands at work had no clear effect on psychological wellbeing in the low work–life imbalance group, they did have an effect in the high work–life imbalance group. This finding suggests that, when work–life imbalance is already high, psychological wellbeing decreases as demands at work increase. When work–life imbalance is low, demands at work ultimately do not impact presenteeism. Work–family conflict is an important factor that can affect psychological wellbeing (the WHO-5 index) and mental health [19,39]. In a qualitative study on work–life balance among nurses with children, positive keywords, such as “the reason I can go on between work and child-rearing” and “finding hope between work and child-rearing”, were identified [34]. Focusing on “finding hope between work and child-rearing,” the study found that the participants, who were nurses, wanted to feel pride in their profession, and tended to be proactive about their life and do their best to balance work and family [34]. In addition, according to a study of Polish nurses, stress from work overload negatively affected nurses’ mental health. However, increasing nurses’ sense of responsibility for their work had a positive effect on their mental health [40]. Among female workers in the health and social work fields who were participants in this study, an appropriate amount of work appeared to increase psychological wellbeing, reflecting the desire of the participants to demonstrate work competency as a professional while maintaining work–life balance. Therefore, the active development of strategies to reduce work–life imbalance among female workers in the health and social work sectors is necessary, and efforts to provide continuing education in the workplace should be made to establish an appropriate workload, affirm positive professional identities, and increase competence. More in-depth studies on work–life imbalance, psychological wellbeing, and demands at work are needed in the future to confirm this finding.

The difference in the effect of social community at work on psychological wellbeing was not large between the two groups, and, in both groups, psychological wellbeing was high when social community at work was high. In other words, psychological wellbeing decreases when the sense of social community at work is low. In a study involving teachers, a strong sense of social community at work was found to be an important predictive factor that decreased the risk of depression, even after controlling for sociodemographic factors and other psychological and social factors [33].

The effect of trouble sleeping on fatigue and muscle aches was greater in the low work–life balance group. When sleep quality is poor, one may experience more fatigue due to the difficulty of physical recovery brought on by a lack of sleep [20]. Trouble sleeping was expected to have a greater impact on fatigue and muscle aches when work–life imbalance was high; however, unexpectedly, trouble sleeping had a greater effect on fatigue and muscle aches in the low work–life imbalance group. More in-depth studies on work–life imbalance, trouble sleeping, and fatigue and muscle aches are needed in the future to confirm and examine this finding.

The effect of psychological wellbeing on fatigue and muscle aches was greater in the high work–life imbalance group. Many previous studies have shown that work–life imbalance had a negative effect on psychological and physical health [1,38,39]. These findings support the results of this study, which found that psychological wellbeing affected fatigue and muscle aches more in the high work–life imbalance group.

The effect of fatigue and muscle aches on presenteeism was greater in the high work–life imbalance group. According to Camerino [3], work–life imbalance is associated with presenteeism. This finding supports the results of this study, which found that fatigue and muscle aches had a greater impact on presenteeism in the high work–life imbalance group.

To summarize the above results, it was found that demands at work did not influence psychological wellbeing in the path model of the low work–life imbalance group. This finding suggests that demands at work ultimately do not influence presenteeism when work–life imbalance is low. The effects among variables were also smaller in the low work–life imbalance group. This result may indicate the importance of strategies to mitigate work–life imbalance.

## 5. Limitations

The limitations of this study are as follows. First, the subjects of this study were workers drawn from a comprehensive sample of occupational settings in the health and social work sectors. Therefore, in the future, it is suggested that follow-up studies target workers in specific sectors. Second, this study explained presenteeism using a limited number of factors, since it was based on secondary data from the KWCS. However, this study is meaningful, since it confirmed the mediating effects of psychological health issues (trouble sleeping and psychological wellbeing) and physical health issues (fatigue and muscle aches) in the relationship between work characteristics (demands and social community at work) and presenteeism among female workers in the health and social work sectors. Another meaningful finding is that these variables impacted presenteeism more when work–life imbalance was high.

## 6. Conclusions

This multigroup path analysis study was conducted to examine the effects of work characteristics related to work–life imbalance on presenteeism among female workers in the health and social work sectors.

In the path model with the full sample, presenteeism was found to be high when demands at work were high and a sense of social community at work was low, mediated by trouble sleeping, psychological wellbeing, and fatigue and muscle aches. In the path model of the low work–life imbalance group, demands at work did not have a direct effect on psychological wellbeing, while there was a direct effect in the path model of the high work–life imbalance group.

Based on the results of this study, the following suggestions are made. First, human resources departments and managers that oversee on-site personnel in health and social work fields should recognize the relationships between work characteristics (demands and social community at work), mental health issues (sleeping trouble and psychological wellbeing), and physical health issues (fatigue and muscle aches) that influence presenteeism. Second, intervention programs should be developed to assess and prevent mental health issues (sleeping trouble and psychological wellbeing) and physical health issues (fatigue and muscle aches) due to work characteristics (demands at work and social community at work) before they arise in order to increase the effectiveness of such programs. Third, the results of this study pertain to presenteeism among female workers in the health and social work sectors, so we hope that future studies can expand the scope of research to include female workers in a wider variety of sectors and conduct comparative analyses based on the results of this study.

## Figures and Tables

**Table 1:** Correlations between variables.

Variables	1	2	3	4	5	6	7
1. Demands at work	1						
2. Social community at work	0.04 *	1					
3. Trouble sleeping	0.22 ***	−0.10 ***	1				
4. Psychological wellbeing	0.08 ***	0.32 ***	−0.11 ***	1			
5. Fatigue and muscle aches	0.02	−0.03	0.15 ***	−0.22 ***	1		
6. Presenteeism	0.00	−0.04	0.12 ***	−0.12 ***	0.34 ***	1	
7. Work–life imbalance	0.32 ***	−0.06 **	0.32 ***	−0.01	0.04 *	0.11 ***	1

* *p* < 0.05, ** *p* < 0.01, *** *p* < 0.001.

**Table 2:** The results of the path analysis.

Path			B	SE	*β*	C.R.	*p*	R^2^
Demands at work	→	Trouble sleeping	0.160	0.015	0.219	10.611	<0.001	0.061
Social community at work	→	Trouble sleeping	−0.157	0.027	−0.122	−5.901	<0.001	
Demands at work	→	Psychological wellbeing	0.158	0.033	0.096	4.793	<0.001	0.109
Social community at work	→	Psychological wellbeing	0.909	0.058	0.314	15.607	<0.001	
Trouble sleeping	→	Fatigue and muscle aches	0.083	0.012	0.136	6.622	<0.001	0.078
Psychological wellbeing	→	Fatigue and muscle aches	−0.062	0.006	−0.231	−11.240	<0.001	
Fatigue and muscle aches	→	Presenteeism	0.171	0.010	0.339	16.938	<0.001	0.115

**Table 3:** Indirect effects of independent variables on presenteeism.

Path	Indirect Effect	SE	95% CI		*p*
			LLCI	ULCI	
Demands at work → Trouble sleeping → Fatigue and muscle aches → Presenteeism	0.0023	0.0005	0.0015	0.0033	<0.001
Demands at work → Psychological wellbeing → Fatigue and muscle aches → Presenteeism	−0.0017	0.0004	−0.0026	−0.0010	<0.001
Social community at work → Trouble sleeping → Fatigue and muscle aches → Presenteeism	−0.0022	0.0006	−0.0037	−0.0013	<0.001
Social community at work → Psychological wellbeing → Fatigue and muscle aches → Presenteeism	−0.0097	0.0014	−0.0128	−0.0072	<0.001

**Table 4:** The results of the path analysis according to work–life imbalance.

Path			Work–Life Imbalance						Critical Ratios
			Low			High			
			*β*	C.R.	*p*	*β*	C.R.	*p*	
Demands at work	→	Trouble sleeping	0.067	2.196	0.028	0.222	7.748	<0.001	5.552
Social community at work	→	Trouble sleeping	−0.070	−2.307	0.021	−0.136	−4.739	<0.001	−2.432
Demands at work	→	Psychological wellbeing	−0.014	−0.478	0.633	0.198	7.122	<0.001	7.600
Social community at work	→	Psychological wellbeing	0.342	11.881	<0.001	0.280	10.067	<0.001	−1.814
Trouble sleeping	→	Fatigue and muscle aches	0.213	7.198	<0.001	0.078	2.750	0.006	−4.448
Psychological wellbeing	→	Fatigue and muscle aches	−0.179	−6.062	<0.001	−0.271	−9.525	<0.001	−3.463
Fatigue and muscle aches	→	Presenteeism	0.267	9.047	<0.001	0.381	13.905	<0.001	4.858

## Data Availability

Not applicable.
